# The Philosophy of Mystery

**Published:** 1841-10

**Authors:** 


Art. X.—*The Philosophy of Mystery.* By W. C. Dendy, Surgeon of the Royal Infirmary for Children, &c.—*London,* 1841. 8vo, pp. 434.

The main object of this volume is to give an account, and, as far as may be, a rational explanation, of the mysterious subjects of dreams, ghosts, visions, prophetic warnings, &c. &c., and it must be admitted that the author, in his attempts to attain it, has contrived to write a very clever and most amusing book. Amid the mass of scientific and practical matter that calls for notice on all sides, we cannot devote sufficient space to do justice to Mr. Dendy's elegant production; we shall therefore pass it by with the general remark that its perusal will both gratify and instruct all readers of taste, especially those who, like the author, have at once a turn for poetry and metaphysics. Mr. Dendy's industry and erudition are equally conspicuous with his talents in this production, which comprehends a mass of fantastic legendary lore of astonishing extent and variety. We think, indeed, if the book has any fault it is in its over-copiousness of illustration, and occasional diffuseness both of method and style: it is, however, on the whole, extremely creditable to Mr. Dendy, and cannot fail to give him an honorable place among our literary writers.

